# Data on characterization of magnetic nanoparticles stabilized with fusion protein of Barstar and C-term part of Mms6

**DOI:** 10.1016/j.dib.2018.10.173

**Published:** 2018-11-03

**Authors:** Victoria O. Shipunova, Polina A. Kotelnikova, Ulkar F. Aghayeva, Oleg A. Stremovskiy, Alexey A. Schulga, Maxim P. Nikitin, Sergey M. Deyev

**Affiliations:** a Shemyakin–Ovchinnikov Institute of Bioorganic Chemistry, Russian Academy of Sciences, 16/10 Miklukho–Maklaya Street, Moscow 117997, Russia; b National Research Nuclear University MEPhI, Kashirskoe shosse 31, Moscow 115409, Russia; c Moscow Institute of Physics and Technology (State University), 9 Institutskiy per., Dolgoprudny, Moscow Region 141700, Russia

## Abstract

Mms6 is a protein that plays crucial role in the biomineralization and formation of magnetosomes in magnetotactic bacteria Magnetospirillum magneticum (strain AMB-1). We developed a fusion protein of C-term part of Mms6 and Barstar (natural inhibitor of ribonuclease Barnase), namely, Bs-C-Mms6. This protein successfully stabilized uncoated monocrystalline Fe_3_O_4_ magnetite nanoparticles in buffered solutions. Here, we present data regarding the synthesis and characterization of magnetite nanoparticles stabilized with Bs-C-Mms6. For further interpretation of the data presented in this article, please see the research article ‘Self-assembling nanoparticles biofunctionalized with magnetite-binding protein for the targeted delivery to HER2/neu overexpressing cancer cells’ (Shipunova et al., 2018) [1].

**Specifications table**

**Table:** 

Subject area	*Biology.*
More specific subject area	*Nanobiotechnology.*
Type of data	*Tables and images.*
How data was acquired	*Images were acquired with transmission electron microscope JEOL JEM-2100 (JEOL Ltd.) and size distributions were acquired using dynamic light scattering method with Zetasizer Nano ZS analyzer (Malvern Instruments, Ltd.).*
Data format	*Raw and analyzed.*
Experimental factors	*Magnetite nanoparticles were synthesized by coprecipitation of iron salts FeCl*_*2*_*and FeCl*_*3*_*and stabilized with the fusion protein of Barstar and C-term part of Mms6 protein in concentrations ranging from 0.3 µM to 40 µM.*
Experimental features	*The nanoparticle properties were studied by transmission electron microscopy and dynamic light scattering.*
Data source location	*Moscow, Russia.*
Data accessibility	*All data are presented in this article.*

**Value of the data**

• The data demonstrate the significant change in the hydrodynamic size distribution of nanoparticles after the incubation with Bs-C-Mms6 protein.
• The data shows that 5 µM of Bs-C-Mms6 protein is enough to stabilize the uncoated Fe_3_O_4_ magnetic nanoparticles at concentration of 0.5 g/L in water.
• The data may be relevant for other researchers using fusion proteins containing Mms6 for magnetic nanoparticle stabilization, modification and application for cell culture experiments.
• The data may be relevant for further studies that focus on the use of such biofunctionalized nanoparticles for *in vivo* tumor cell targeting.

## 1 Data

In this report, we summarize data on characterization of magnetite nanoparticles stabilized with fusion protein of Barstar and C-term part of Mms6 (Bs-C-Mms6) designed in the study [1]. We studied the morphology of the synthesized nanoparticles with transmission electron microscopy and characterized the change of hydrodynamic size distribution of nanoparticles after the incubation with different concentrations of Bs-C-Mms6 as well as with other non-specific proteins, such as pristine Barstar, Bs and bovine serum albumin, BSA.

## 2 Experimental design, materials and methods

### 2.1 Materials

Sigma, Germany: Iron (III) Chloride hexahydrate (FeCl_3_·6H2O), Iron (II) Chloride tetrahydrate (FeCl_2_·4H_2_O), Ammonium hydroxide solution (NH_4_OH), Nitric acid (HNO_3_). All other chemical reagents were of analytical grade and were used without further purification.

### 2.2 Magnetic nanoparticle synthesis

Nanoparticles of magnetite (MPs) were synthesized similar to described by us earlier [2] with the following modifications. 2.36 g of FeCl_3_·6H_2_O and 0.86 g of FeCl_2_·4H_2_O (2.15 g) were dissolved in 40 mL of MilliQ water. Then, 5 mL of 30% NH_4_OH was added. The solution was heated to 90 °C for 2 h with constant stirring. The resulted magnetite nanoparticles were separated with 30-mm Nd-Fe-B magnet and incubated in 2 M HNO_3_ for 5 min at RT. Then, resulted MPs were sequentially separated from HNO_3_ with MilliQ water. Each separation step included the addition of 10 mL of MilliQ water with the application the magnet to the bottom of the tube for 5 min. The separation steps were repeated 5 times, the fifth fraction was used for further modification.

### 2.3 Nanoparticle characterization with transmission electron microscopy

**Fig. 1:**
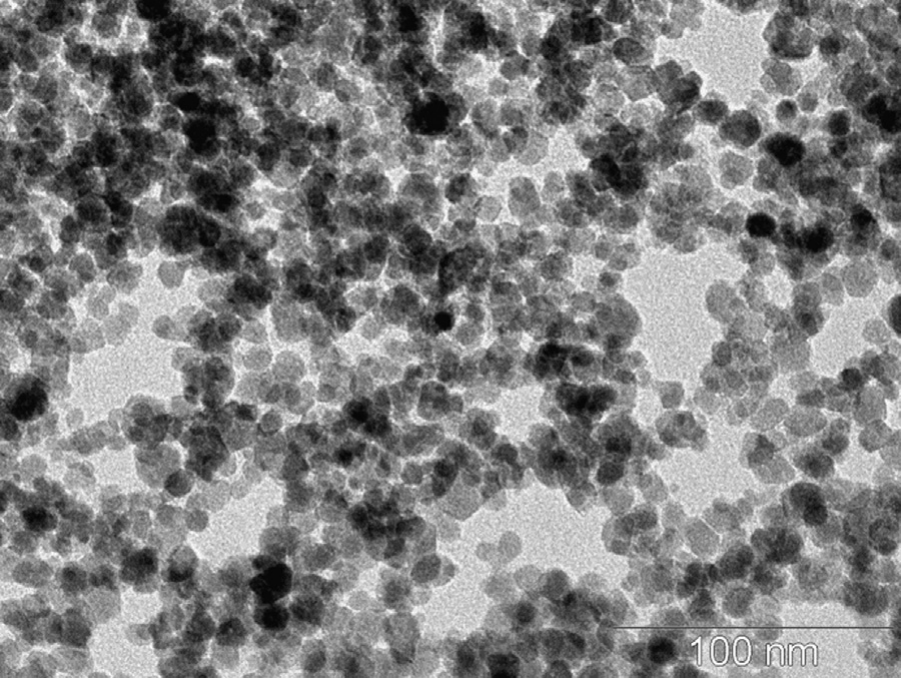
Transmission electron microscopy of magnetite nanoparticles obtained with co-precipitation of iron salts. The particles are characterized with a close to spherical shape and by the mean diameter of 10.9 ± 1.9 nm.

As obtained MPs were studied by transmission electron microscopy at JEM-2100 (JEOL, Ltd.) with an accelerating voltage of 200 kV. The morphology of the obtained MPs corresponds to monocrystalline nanoparticles with a close to spherical shape with the average diameter of 10.9 ± 1.9 nm, the size was obtained as the result of electron microphotograph processing (see Fig. 1).

### 2.4 Dynamic light scattering

The hydrodynamic size of the synthesized MP was measured with Zetasizer Nano ZS analyzer (Malvern Instruments, Ltd.) in water at 25 °C. The measurements showed the hydrodynamic diameter of the particles to be 79.9 nm (PdI width is 31.8 nm). Such a significant difference of TEM physical size and hydrodynamic size could be explained by the formation of the hydration shell on the surface of the particles in water, which significantly affects the dynamic light scattering measurements.

### 2.5 Nanoparticle stabilization with Bs-C-Mms6

Fe_3_O_4_ magnetic nanoparticles obtained as described above were washed trice with MilliQ water and resuspended in MilliQ water to get a final concentration of 0.5*g*/L. Then, MPs were incubated with Bs-C-Mms6 protein (at final concentrations of protein from 0.3 µM to 40 µM) for 8 h at RT with occasional sonication in an ultrasonic bath RK 31H (Bandelin Sonorex) to get MP*Bs-C-Mms6 particles. Next, particles were washed with MilliQ water from non-bound protein by triple centrifugation for 10 min at 20,000*g*, +4 °C in the desktop centrifuge 5430R (Eppendorf). Finally, particles were resuspended in phosphate buffered saline (PBS) in order to study their stability in buffered solutions. Bovine serum albumin (BSA) and Barstar (Bs) proteins were used instead of Bs-C-Mms6 for control experiments to get MP*BSA and MP*Bs particles.

The hydrodynamic size distribution of the modified nanoparticles was measured with Zetasizer Nano ZS in water at concentration of 0.1 g/L. The average sizes (*Z*-average) and PdI widths are presented in Table 1 for all modified nanoparticles.

**Table 1:** Hydrodynamic sizes of Fe_3_O_4_ nanoparticles incubated with Bs-C-Mms6, Bs and BSA proteins.

Particle notation	MP*Bs-C-Mms6		MP*Bs		MP*BSA	
Concentration of protein, µM	*Z*-average	PdI Width	*Z*-average	PdI Width	*Z*-average	PdI Width
40	838.6	440.2	109.5	38.9	103.8	32.9
20	396.8	151.8	137.3	47.1	108.6	32.8
10	352.6	153.2	116.7	41.4	102.8	34.6
5	136.5	38.4	111.0	39.5	96.7	35.4
2.5	126.1	44.9	114.0	33.5	107.6	43.0
1.25	98.6	37.9	86.8	32.5	105.7	43.3
0.625	86.3	31.7	93.8	34.8	85.7	33.8
0.3125	89.8	35.4	72.1	30.1	133.8	77.0
0	79.9	31.8	79.9	31.8	79.9	31.8

**Fig. 2:**
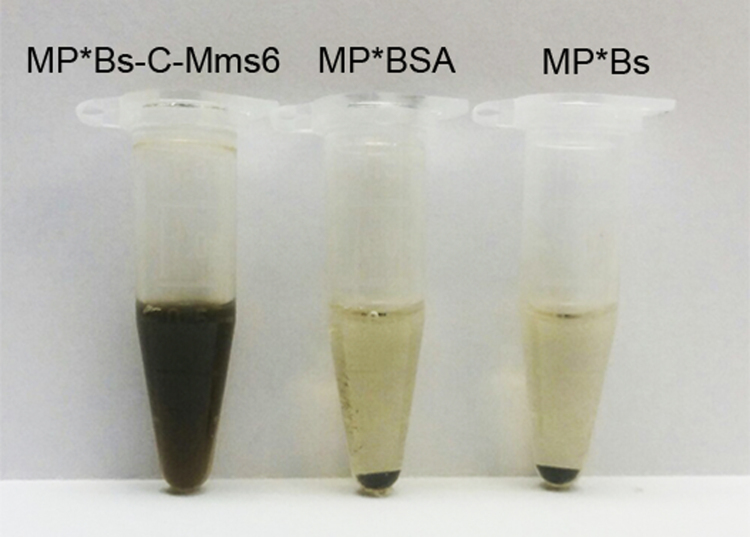
Colloidal stability of magnetite nanoparticles modified with Bs-C-Mms6 protein in phosphate buffered solution (PBS). Particles were incubated with 5 µM of Bs-C-Mms6, Bs or BSA and then transferred to PBS. Only MP*BS-C-Mms6 exhibit excellent colloidal stability in PBS for a long period of time (up to 2 month).

The significant increase in the size of MP*Bs-C-Mms6 can be observed when the nanoparticles were incubated with the Bs-C-Mms6 protein in concentration more than 2.5 µM. However, only particles incubated with Bs-C-Mms6 in concentrations of protein higher than 5 µM exhibited excellent colloidal stability in PBS for a long period of time (2 months, no further observations were made). The use of control proteins, namely, BSA and Bs in the whole range of tested concentrations, did not result in the stabilization of the nanoparticles in the PBS: the particles precipitated in PBS within a few minutes (see Fig. 2).

According to the technology of Nanoparticle Tracking Analysis, NTA (NanoSight NS300, Malvern Instruments Ltd.) the mass concentration of the synthesized MP of 0.5 g/L corresponds to the molar concentration of MP equal to (0.9 ± 0.1)·10^−^^8^ М. This means that approximately 500 molecules of Bs-C-Mms6 protein are enough for stabilization of one nanoparticle in PBS.
